# Supplementary material for: Causal Relationship between Aging and Anorexia Nervosa: A White-Matter-Microstructure-Mediated Mendelian Randomization Analysis
Source: Biomedicines. 2024 Aug 16;12(8):1874. doi: 10.3390/biomedicines12081874 (PMC11351342; doi:10.3390/biomedicines12081874)
Supplement: Supplementary file 1 [file biomedicines-12-01874-s001.zip › Supplementary Figure/Figure S8/Leaveoneout plot of MR analysis for TL to ubm-b-2017.pdf]

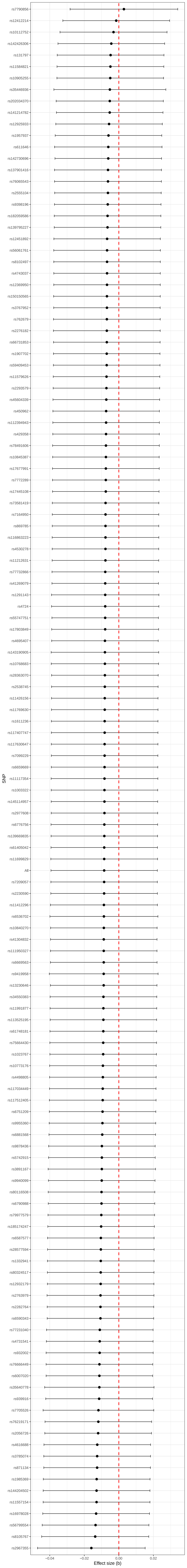

SNP
Effect size (b)
-0.04
-0.02
0.00
0.02
rs7790856
rs12412214
rs10112752
rs142426306
rs131797
rs11584821
rs10905255
rs35446936
rs202034370
rs141214782
rs12925933
rs1957937
rs611646
rs142730696
rs137901416
rs76065543
rs2555104
rs9398196
rs182059586
rs139795227
rs12451892
rs56061761
rs8102497
rs4743037
rs12369950
rs150150565
rs3767952
rs762679
rs2276182
rs66731853
rs1907702
rs59409453
rs11579626
rs2293579
rs45604339
rs450962
rs112394943
rs429358
rs78491606
rs10845387
rs17677991
rs7772289
rs17445108
rs73581419
rs7164950
rs869785
rs116863223
rs4530278
rs11212631
rs77732866
rs41269079
rs1291143
rs4724
rs55747751
rs17803849
rs4695407
rs143190905
rs10768683
rs28363070
rs2538745
rs11426156
rs11769630
rs1611236
rs117407747
rs117630647
rs7099229
rs6659669
rs11117354
rs1003322
rs145114957
rs2977608
rs6776756
rs139669835
rs61405042
rs11699829
All
rs7209057
rs2230590
rs11412296
rs6536702
rs10840270
rs41304832
rs111950327
rs6669563
rs9419958
rs13230646
rs34550383
rs11991877
rs113525195
rs61748181
rs75664430
rs1023767
rs10773176
rs4498805
rs117034449
rs117512405
rs6751209
rs9955360
rs6881568
rs9878436
rs5742915
rs3891167
rs9940099
rs80116508
rs6790988
rs79977579
rs185174247
rs6587577
rs28577594
rs1332941
rs80024517
rs12932179
rs2763979
rs2282764
rs6590343
rs77231040
rs4731541
rs932002
rs76666449
rs6007020
rs35640778
rs939916
rs7705526
rs76219171
rs2056726
rs4616688
rs3785074
rs871134
rs1985369
rs144204502
rs11557154
rs16978028
rs56799554
rs8105767
rs2967355
